# AAPM medical physics practice guideline 13.a: HDR brachytherapy, part A

**DOI:** 10.1002/acm2.13829

**Published:** 2023-02-20

**Authors:** Susan L. Richardson, Ivan M. Buzurovic, Gil'ad N. Cohen, Wesley S. Culberson, Claire Dempsey, Bruce Libby, Christopher S. Melhus, Robin A. Miller, Daniel J. Scanderbeg, Samantha J. Simiele

**Affiliations:** ^1^ Swedish Medical Center Seattle Washington USA; ^2^ Brigham & Women's Hospital, Harvard Medical School Boston MA USA; ^3^ Memorial Sloan‐Kettering Cancer Center New York NY USA; ^4^ University of Wisconsin‐Madison Madison WI USA; ^5^ Calvary Mater Newcastle Hospital University of Newcastle, Callaghan, Australia University of Washington Seattle USA; ^6^ Moffitt Cancer Center Tampa FL USA; ^7^ Tufts Medical Center Tufts University School of Medicine Boston MA USA; ^8^ Multicare Regional Cancer Center Northwest Medical Physics Center Tacoma WA USA; ^9^ UC San Diego California USA; ^10^ University of Texas MD Anderson Cancer Center Houston TX USA

**Keywords:** brachytherapy, HDR, MPPG, practice guideline

## Abstract

The American Association of Physicists in Medicine (AAPM) is a nonprofit professional society whose primary purposes are to advance the science, education, and professional practice of medical physics. The AAPM has more than 8000 members and is the principal organization of medical physicists in the United States.

The AAPM will periodically define new practice guidelines for medical physics practice to help advance the science of medical physics and to improve the quality of service to patients throughout the United States. Existing medical physics practice guidelines (MPPGs) will be reviewed for the purpose of revision or renewal, as appropriate, on their fifth anniversary or sooner.

Each medical physics practice guideline represents a policy statement by the AAPM, has undergone a thorough consensus process in which it has been subjected to extensive review, and requires the approval of the Professional Council. The medical physics practice guidelines recognize that the safe and effective use of diagnostic and therapeutic radiology requires specific training, skills, and techniques, as described in each document. Reproduction or modification of the published practice guidelines and technical standards by those entities not providing these services is not authorized.

The following terms are used in the AAPM practice guidelines:

(1) Must and must not: Used to indicate that adherence to the recommendation is considered necessary to conform to this practice guideline.
(2) Should and should not: Used to indicate a prudent practice to which exceptions may occasionally be made in appropriate circumstances.

Approved by AAPM's Executive Committee April 28, 2022.

## 1 DEFINITIONS AND ACRONYMS

**ABR**: American Board of Radiology.

**ABS**: American Brachytherapy Society.

**ACR**: American College of Radiology.

**AMP**: authorized medical physicist—an individual who meets the requirements listed in 10 CFR § 35.

**ASTRO**: American Society for Radiation Oncology.

**AU**: authorized user—a physician who meets the requirements listed in 10 CFR § 35 or is identified as an AU on a license or permit regarding medical use of byproduct material.

**CBCT**: Cone Beam Computed Tomography.

**CFR**: Code of Federal Regulations.

**COMP**: Canadian Organization of Medical Physicists.

**CPQR**: Canadian Partnership for Quality Radiotherapy.

**Dosimetrist**: a qualified medical dosimetrist as defined by the Association of Medical Dosimetrists as “an individual who is competent to practice under the supervision of a qualified physician and qualified medical physicist.”

**ESTRO**: European Society for Therapeutic Radiology and Oncology.

**GEC**: The Groupe Européen de Curiethérapie.

**HDR**: high dose‐rate brachytherapy—refers to dose rates higher than 12 Gy/h (ICRU38^1^).

**IAEA**: International Atomic Energy Agency.

**ICRP**: International Commission on Radiological Protection.

**IFU**: instructions for use—instructions for use provided from the manufacturer of applicators or devices.

**IPEM**: Institute of Physics and Engineering in Medicine.

**IORT**: Intraoperative Radiotherapy.

**NCRP**: National Council on Radiation Protection and Measurements.

**NRC**: Nuclear Regulatory Commission.

**PMI**: preventative maintenance inspection.

**QA**: quality assurance—as defined in the AAPM Task Group 100 report: “QA confirms the desired level of quality by demonstrating that the quality goals for a task or parameter are met.”

**QC**: quality control—as defined in the AAPM Task Group 100 report: “QC encompasses procedures that force the desirable level of quality by evaluating the current status of a treatment parameter, comparing the parameter with the desired value, and acting on the difference to achieve the goal.”

**QM**: quality management—as defined in the AAPM Task Group 100 report: “QM consists of all the activities designed to achieve the desired quality goals.”

**QMP**: qualified medical physicist—as defined by AAPM Professional Policy 1.

**RAM**: radioactive material.

**TGT**: transfer guide tube.

## 2 INTRODUCTION

The goal of this report is to assist the clinical medical physicist in assuring that key quality metrics and practice considerations are met to ensure the safe, reliable, and reproducible application of high‐dose rate (HDR) brachytherapy. This guideline has been developed to provide appropriate minimum standards for such services. The secondary goal is to provide recommendations to the regulatory community from the experts on this practice guideline to guide the adoption of regulations in the future. This MPPG is limited to iridium‐192‐based HDR brachytherapy and will not discuss electronic, low‐dose rate, pulsed dose rate brachytherapy, or any alternative radionuclides.

### 2.1 Scope

This report has been divided into two parts. Part A describes the infrastructure and program design in the creation of an afterloader‐based HDR brachytherapy program. Part B (a separate, subsequent report) describes the clinical treatment processes including imaging, planning, and treatment delivery.

### 2.2 Disclaimer

It is the responsibility of all healthcare staff to be familiar with state and federal guidelines that may take precedence over American Association of Physicists in Medicine (AAPM) recommendations that are provided in this report. Each health care facility may have site‐specific or state‐mandated needs and requirements that may modify their usage of these recommendations.

### 2.3 Background

Brachytherapy enjoys a long and rich history that transcends the practice of radiation therapy. Shortly after the first observations of self‐inflicted biological effects by Henri Becquerel and Pierre Curie, the first encapsulated radium source was provided by Pierre and Marie Curie to Henri‐Alexandri Danlos in Paris (1903) for dermatological therapies. Over a century of advances and development followed this first implementation of radiation therapy.^2^ The modern nuclear era, including human‐induced radioactivity, and the advent of the computer age allowed brachytherapy to transform from a manually delivered qualitative practice to an automated, quantitative one. Mechanical advances in remote source afterloading provided significant radiation dose reduction to providers. Additionally, the preference to reduce in‐patient stays, which had a concomitant need for expensive, shielded medical units, led to the advent of HDR brachytherapy. Similar to how intensity‐modulated radiation therapy (IMRT) advanced external beam radiation therapy in the 1990s, HDR brachytherapy was the high‐tech treatment modality that advanced the field of brachytherapy in the 1980s. However, many of the reported drivers of HDR brachytherapy at the time were socio‐economical. Similar to IMRT, HDR brachytherapy lacked prospective clinical trials to demonstrate the clinical benefits and questions regarding dose, fractionation, and their related radiobiological considerations were expected to take years to answer.^3^

Today, HDR brachytherapy is a commonly used therapeutic technique. It is a resource‐intensive modality with an oversight by applicable government regulation and recommended practices by professional societies, accreditation standards, and many others. The following section provides an overview of guiding regulations, clinical practice recommendations, and manufacturers’ responsibilities that are applicable to the practice of HDR brachytherapy.

## 3 REGULATORY REQUIREMENTS

The Code of Federal Regulations (CFRs) are general rules applied nationally and are organized under the United States president through the executive branch. Regulatory responsibility for radioactive material is the responsibility of the Nuclear Regulatory Commission (NRC) and is listed as Title 10 in the CFRs. Federal law allows states to administer their own regulatory programs so long as they meet or exceed the requirements of the CFRs. These agencies are subject to periodic review by the NRC to maintain Agreement State status. Medical physicists practicing in agreement states must review their state regulations as they may differ from the federal ones.

The NRC oversees the licensing of all naturally occurring or accelerator‐produced materials (NARM) including nuclear reactor‐produced materials. This latter material is known as byproduct material. Title 10 CFR §37 describes physical protection of *Category 1* and *Category 2* quantities of radioactive material (RAM). NRC defines these sources as “risk‐significant sources” and they are listed in an IAEA publication.^4^ Most users will not trigger Category 2 requirements of greater than 21.6 Ci or 799.2 GBq (for Ir‐192) of contained activity unless they have multiple afterloaders. However, newer afterloaders have better shielding and higher activity sources (10–15 Ci), so each facility is responsible for evaluating their total on‐site activity with regards to the security of their sources and licensing requirements.

Due to potential variations in specific rules for the current agreement states, the regulations in the federal register (i.e., the CFRs), which represent minimum compliance expectations, will be discussed in the subsequent sections (I–VIII) where appropriate. Table 1 summarizes the legal references and topics discussed. When readily available, international publications have also been listed. All CFR reports can be accessed at nrc.gov.

**TABLE 1:** Regulations and supporting references for implementation of an HDR brachytherapy program

**Regulations**	**References**	**Topics covered**
RAM licensing	10 CFR § 30 10 CFR § 33	The process by which an organization or individual may receive a license entitling them to receive, possess, use, transfer, or deliver RAM
Personnel monitoring	10 CFR § 20 IAEA safety standards no. GSR Part 3^8^ NCRP report no. 116^9^	Standards of protection for the public against exposure to radiation and the limits of exposure for radiation workers
Shielding	NCRP report no. 49^5^ IPEM report 75^10^ IAEA safety report series 47^11^	Guidance on shielding design
Security	10 CFR § 37	Specifies the requirements for physical protection of large quantities of radioactive material
	10 CFR § 20.1801 IAEA nuclear security series no. 11‐G^12^	Specifies security of stored materials
	10 CFR §35.610	Specifies security of HDR hardware and computers
Transportation and handling	49 CFR § 173 IAEA safety standards no. SSR‐6 (Rev. 1)^13^	General transportation requirements for RAM
	10 CFR § 71	Packaging, shipment, and transport of RAM
	10 CFR § 20.1906	Receiving and opening of RAM
	49 CFR § 172	Receiving or packaging RAM
Records	10 CFR § 30.51 10 CFR § 40.61	Receipt, inventory, acquisition, transfer, and disposal
Periodic spot checks	10 CFR § 35.643 ICRP publication 97^14^	Periodic spot checks for remote afterloader units
Training	10 CFR § 19 ICRP publication 97	Notices, instructions, and reports by licensees and regulated entities to RAM workers
Patient treatment	10 CFR § 35.615	AMP presence during treatment
	10 CFR § 35.604	Radiation surveys
	10 CFR § 35.610 ICRP publication 97	Emergency Procedures

Abbreviations: AMP, authorized medical physicist; CFR, code of federal regulations; IAEA, International Atomic Energy Agency; ICRP, International Commission on Radiological Protection; IPEM, Institute of Physics and Engineering in Medicine; NCRP, National Council on Radiation Protection and Measurements; RAM, radioactive material.

Beyond the borders of the United States, most sovereign nations have implemented regulations to guide the use of radioactive materials. In support of the peaceful use of atomic energy, the International Atomic Energy Agency (IAEA) was founded by the United Nations in 1957. It provides guidance and technical cooperation for 172 member nations and partners worldwide. Its primary mission is to promote safe, secure, and peaceful nuclear technologies. In this light, the IAEA assists in defining technical standards for the use of radioactive and byproduct material some of which are listed in Table 1.

### 3.1 RAM licensing

Any healthcare facility offering brachytherapy services must have a radioactive materials license that allows them to receive, possess, utilize, and transport such sources. This license becomes a mechanism by which the regulatory agency can supervise the use of radioactive source and byproduct material and ensure that licensees comply with applicable regulations.^5^

### 3.2 Personnel monitoring

Personnel monitoring for radiation workers is only required if there is an expectation that staff receives greater than 10% of the regulatory limits; however, brachytherapy providers should be actively monitored in the event of an emergency response.

### 3.3 Shielding

There are no specific US regulations regarding shielding design or requirements, with the exception that shielding must be installed to ensure that the requirements for radiation exposure to personnel and members of the public in 10 CFR § 20 are met. National Council on Radiation Protection and Measurements (NCRP) report No. 49 offers guidance on structural shielding design for gamma rays up to 10 MeV, which would include iridium‐based HDR. A regulatory agency may require review and approval of shielding plans prior to the construction of a new facility or modification of an existing one.

### 3.4 Security

Source security is defined as a set of measures required to prevent unauthorized access, damage, loss, theft, or unauthorized transfer of radioactive sources. NRC and Agreement States established “a multilayered, comprehensive security program” to protect these sources. The licensee must generate policies and procedures that govern the storage and transfer of radioactive material to ensure compliance with this standard as per 10 CFR § 37. For example, designated storage areas and enhanced security measures may be helpful for compliance. Regarding source receipt, the licensee is expected to expeditiously take possession of the package. This should require creating a lockable storage area where packages are received before they can be surveyed and transferred to a secure storage area.

### 3.5 Transportation regulations

Shippers and transporters must receive specialty training in these regulations every 3 years to assure compliance (see 10 CFR §172). Licensees must establish processes to comply with specific requirements around the receipt of RAM. Licensees must perform a wipe test within 3 h of the receipt of normal form RAM during business hours (or by the beginning of the next business day if delivered after hours) to assure that there were no leaks or spills of the material in transit by examining the packing for contamination. Some HDR sources are sent as special form RAM and may be exempted from wipe testing requirements (see 10 CFR § 20.1906). The AAPM virtual library contains two excellent overviews of this training.^6^

### 3.6 Records

The current regulations regarding source or by‐product material state that the records must be maintained for the receipt, duration of possession, and the transfer or disposition of the material for three years. Additionally, records for spots checks and surveys must be kept for 3 years. More details can be found in 10 CFR § 30.51 and 10 CFR § 40.61.

### 3.7 Periodic spot checks

This section will be addressed in Section 7.1.

### 3.8 Training

Training of staff will be addressed in Section 6.2.

### 3.9 Patient treatment

Due to the rate of dose delivery in HDR brachytherapy, an authorized medical physicist (AMP) and authorized user (AU) must be physically present for the initiation of all patient treatments. The AMP must remain immediately available for the entire duration of treatments; a physician with training in emergency procedures may replace the AU for the remaining duration of the treatments. The interpretation of physical presence was later clarified as being within hearing distance of normally spoken voice.^7^ Additionally, AMPs and other involved personnel must participate initially and annually in an emergency drill. While most HDR brachytherapy systems do not merit the enhanced physical security requirements listed in 10 CFR § 37, if the licensee chooses to implement enhanced security practices (e.g., electronic door locks with biometric access) then the medical physicist must evaluate their potential role in an emergency response to assure that the patient can be quickly reached in the event of a system failure (e.g., power loss) or medical emergency (e.g., a cardiac arrest). More information regarding these topics will be given in Part B of this practice guidance report.

## 4 CLINICAL PRACTICE RECOMMENDATIONS

### 4.1 Accreditation standards

There are multiple groups that provide practice accreditation to hospitals and free‐standing radiation therapy clinics. The accreditation may be used as a demonstration of the ability to meet specified standards and may be used in advertising efforts. Accreditation may be obtained by the American College of Radiology (ACR), the American Society for Radiation Oncology (ASTRO) through the Accreditation Program for Excellence (APEx), or through the American College of Radiation Oncology (ACRO). To receive accreditation, sites must demonstrate compliance with the accreditation standards set by the various organizations. At present, there is no regulatory requirement or implication for accreditation.

### 4.2 Professional societies

As a service to their members and to protect and benefit members of the public, professional societies may prepare guidance documents. The AAPM has produced over 100 guidance reports on a variety of topics including HDR brachytherapy, and other aligned societies have also published guidelines and recommendations for HDR brachytherapy including ASTRO, ACR, American Brachytherapy Society (ABS), and European Society for Therapeutic Radiology and Oncology (ESTRO). A wide variety of international entities have also generated guidelines that may be useful. A summary of documents that may be of use to HDR practitioners is found in Table 2.

**TABLE 2:** Selected societal guidance documents on HDR brachytherapy

**Topic**	**Reference**	**Year published**
General HDR brachytherapy QA/QC/QM programs	AAPM report 41^15^	1993
	AAPM report 46^16^	1994
	AAPM report 59^17^	1997
	AAPM report 61^18^	1998
	ASTRO/PRO special article^19^	2014
	IAEA 2D to 3D^20^	2015
	COMP/CPQR quality guidelines^21^	2018
	NCS code of practice^22^	2018
	ACR/ABS/ASTRO practice parameter^23^	2020
	ACR/AAPM technical standard^24^	2020
Dosimetric formalisms and consensus data	AAPM and ESTRO report 229^25^	2012
Uncertainties in brachytherapy	AAPM/GEC‐ESTRO report 138^26^	2011
	GEC‐ESTRO/AAPM review^27^	2014
Treatment planning	AAPM report 62^28^	1998
	CPQR quality guidelines^29^	2018
Model‐based dose calculation	AAPM report 186^30^	2012
Surface brachytherapy	AAPM/GEC ESTRO report 253^31^	2020
Safety and risk analysis methodology	ICRP prevention of accidents^32^	2005
	AAPM report 283^33^	2016
	ASTRO safety is no accident^34^	2012 and 2019

Abbreviations: AAPM, American Association of Physicists in Medicine; ABS, American Brachytherapy Society; ACR, American College of Radiology; ASTRO, American Society for Therapeutic Radiation Oncology; COMP, Canadian Organization of Medical Physicists; CPQR, Canadian Partnership for Quality Radiotherapy; ESTRO, European Society for Therapeutic Radiology and Oncology; GEC, The Groupe Européen de Curiethérapie; HDR, high‐dose rate brachytherapy; ICRP, International Commission on Radiological Protection; NCS, Nederlandse Commissie voor Stralingsdosimetrie; QA, quality assurance; QC, quality control; QM, quality management.

The AAPM has published numerous reports that address HDR brachytherapy in a variety of ways. Several reports include quality assurance (QA) recommendations for remote afterloaders, sources, applicators, and treatment planning systems (TPS). These form the basis for most institution‐specific QA programs. Of note, the most recent of these reports was published in 1998, showing that it has been nearly 20 years since quantitative QA performance benchmark recommendations were defined by the AAPM for brachytherapy. Report 283 (known as the report of TG‐100) introduced the concept of risk‐analysis methods in the formation of quality management (QM) protocols. There are also educational resources from the AAPM Brachytherapy Summer School publications from 1994, 2005, 2013, and 2017, which cover a wide variety of topics.

Clinicians may also refer to other national professional societies to draw from their experience and benefit from their recommendations such as ESTRO and Canadian Organization of Medical Physicists (COMP). These groups have sponsored a large number of publications that may be of interest to HDR brachytherapy physicists. COMP has published recent quality control (QC) guidelines for remote afterloaders, among other pertinent recommendations. Helpful guidance documents may also be found by other national organizations, such as the Netherlands Commission on Radiation Dosimetry group and the Australasian College of Physical Scientists and Engineers in Medicine, among others.

While hundreds of guidance documents may inform readers, health care facilities should follow their own internally defined and approved practices. Internal policy should outline key rules and requirements, while an associated procedure should describe the steps to ensure that policy goals are met.

### 4.3 Manufacturers

Vendors that market and sell medical equipment in the United States must comply with Food and Drug Administration (FDA) regulations. The FDA is organized under the Department of Health and Human Services in the executive branch. Regulations governing the lifecycle of medical devices are located in Title 21 of the CFR, from preclinical use to labeling requirements. Medical device vendors have a responsibility to inform users of issues identified with a specific medical device. These typically take place as a Notice to Users or a Field Change Order in the event that service is required, and the notices may require acknowledgment of receipt by the end user. Users may be required to provide information and access to a vendor in the event of a medical device malfunction. Maintaining contact with the vendor, for example, through a service contract, ensures that users receive critical notifications and safety upgrades and that preventative maintenance is performed as recommended. Users should also ensure they have current copies of manufacturers’ instructions for use (IFU) that define proper use, sterilization requirements (if applicable), and the product lifecycle.

## 5 FACILITY

### 5.1 Vaults

The most common location of an HDR brachytherapy afterloader is in a dedicated vault or suite in which the unit is stored permanently and the patient is treated. An alternative is to use another pre‐existing shielded area of the hospital, such as a linac or imaging vault. Depending on the types of procedures performed at a facility, HDR brachytherapy in an intraoperative radiotherapy (IORT) environment may require treatment in another department's operating room. Each solution presents its own unique challenges and benefits.

#### 5.1.1 Dedicated suites

A dedicated suite is the easiest solution as the patient may wait in the vault during treatment planning and there may be no competing procedures which require the movement of the patient. Equipment storage is usually available through built‐in cabinetry allowing QA devices, applicators, and transfer guide tubes (TGTs) to be stored near the treatment area. Shielding should be designed specifically for HDR radionuclide energy ranges and is generally less costly than shielding for a linac vault. In the design phase, care should be taken to evaluate patient procedures that require ancillary devices and equipment such as overhead lighting, surgical lighting, access to oxygen and anesthesia equipment, radiographic needs, patient monitoring, and so forth. A wide maze and doorway will facilitate patient transport on a gurney.

#### 5.1.2 Shared suites

Due to space limitations, especially within an existing facility, it may not be possible to have a dedicated HDR brachytherapy suite. While this obviates the need to shield a room specifically for brachytherapy, regulations require the presence of interlocks to prevent the accidental use of the linac or simulator during a brachytherapy treatment (and vice versa). While most linac vaults provide adequate shielding for a new HDR brachytherapy source, a survey for hot spots or shielding defects should still be performed.^35^ Additionally, the afterloader must be properly stored and secured in the treatment vault to comply with any state or federal regulations. If shared with a simulator room to facilitate imaging, the patients should be imaged and treated on a non‐radiopaque table. Retrofitting an imaging suite for brachytherapy may be expensive due to shielding requirements. Information on the implementation of HDR brachytherapy in a limited resource setting can be found elsewhere.^36^

#### 5.1.3 Operating room settings

Intraoperative procedures where applicator insertion, imaging, planning, and treatment are performed in one session under anesthesia are becoming increasingly common. Situating an afterloader in an operating room can allow for efficient treatment at the time of surgery, but can introduce other complicating factors such as increased training of nonradiation oncology personnel, the need to interlock and shield multiple doors, addition of warning lights and radiation monitors, and storage and security of the afterloader.^37^ In the rare event of an emergency, the patient may be under anesthesia and dependent on life support equipment and may not be able to be moved out of the procedure or operating room. Instead, the HDR afterloader (or source) must be isolated from the patient. This can be achieved if a small shielded enclosure is constructed as part of the room and serves both as an emergency container of the source and attached applicator(s) and as a secured routine storage area of the afterloading unit.^38^ Additional challenges of this environment include high‐pressure treatment planning time constraints as anesthesia duration should be minimized, and operating room time is costly.

#### 5.1.4 Mobile HDR units

It is possible to transport an HDR unit between multiple locations or to house an HDR unit in a shielded van. This may increase the ability to treat patients who otherwise could not travel for treatment. Ten CFR §35.2080 covers mobile medical services and is not be discussed further in this report.

A summary table of advantages and challenges of various facility types with optional imaging devices is shown in Table 3 below:

### 5.2 Imaging resources

In order to appropriately plan the HDR treatment, the patient should be imaged with the applicator in situ. Placement of the afterloader within a linear accelerator treatment vault can permit the use of the linac's imaging capabilities such as kV imaging and CBCT. Dedicated brachytherapy suites can use a variety of imaging devices, such as a portable CT scanner, portable C‐arm, CT‐on‐rails, MR, or even an MR‐on‐rails. Both surface and intracavitary ultrasound can be utilized to assist in applicator placement as well as being used for planning, such as in HDR prostate brachytherapy. The most common setting is a departmental CT scanner with patient transfer to the HDR treatment room. The physicist should make best use of the imaging resources available. For example, obtaining an MR scan during the course of cervical brachytherapy can be performed at a scanner located outside of the department either with or without the applicator in place.^39^, ^40^ More information about treatment planning imaging and options will be provided in Part B of this report.

### 5.3 Patient management

In addition to the radiotherapy and imaging resources, patients need to be medically managed. Use of anesthesia or conscious sedation requires independent monitoring of the patient. If the patient is to be treated while under anesthesia, vital signs must be monitored from outside the treatment vault. Typical installations will include two independent cameras with one that can be fixed onto the anesthesia equipment for monitoring. Patients can also be medicated orally for pain and anxiety relief, which does not require additional monitoring of the patient by trained personnel.

### 5.4 Transportation and immobilization

The absence of dedicated treatment suites with in‐room imaging requires patients to be transported from the area of applicator placement and/or imaging to the treatment area. This can involve many separate movements of the patient, especially if the applicator is placed in an operating room. Proper training of staff as well as dedicated equipment to move the patient can help mitigate applicator or needle migration or displacement. If possible, the applicator should also be fixed in place with respect to the patient through the use of external fixation. Options include external fixation devices, special brachytherapy underwear, and homemade devices. Interstitial templates may be sutured to the patient, and glue or dental putty may be used to keep catheters and needles in place. Commercial patient transport systems for the movement of brachytherapy patients can be helpful. Regardless of the immobilization and transport method, the patient should be imaged as close to treatment initiation as possible to confirm applicator positioning.

When the HDR unit is located in a facility that is not attached to the health care facility where the applicator is placed, an ambulance service may be necessary to transport the patient. Regardless of the distance involved, this type of transport can pose some challenges: coordination and timing of transportation, support staff for transportation depending on pain control methodology, type of anesthesia used for applicator placement, and applicator movement during transfer.

**TABLE 3:** Summary of facility types for HDR brachytherapy treatments

Location	Advantage	Challenge	Imaging devices
Linac or simulator vault	Existing shielding and space, minimization of patient transport after imaging	Storage, patient scheduling, hardware interlocks	CT CBCT kV imaging ultrasound
Dedicated suite (brachy only)	Access, storage, patient timing	Shielding cost, space limitations, patient transportation if no in‐room imaging	CT Portable CT CT on rails CBCT MR kV imaging ultrasound
Operating room	Access	Shielding, storage, multiple interlocks	Variable

## 6 STAFFING

### 6.1 Participants

The brachytherapy treatment team may consist of a multitude of different members including AU, resident physician, AMP, physics resident, dosimetrist, nurse, therapist, and interdepartmental members like a breast surgeon or anesthesiologist. Together, this team should be informed about the particular patient and work in a collaborative manner toward the ideal patient treatment. This requires good communication and standardization of policy and procedures.

The radiation oncologist is generally present, and the physicist or dosimetrist should be present during the placement of the treatment applicator. The presence of interdepartmental personnel for applicator placement will depend upon the policies and procedures at each health care facility and the complexity of the patient treatment. Staffing needs may vary based on the type of sedation used, such as full or conscious sedation. Members outside of the radiation oncology team who may be needed for certain procedures include, but are not limited to anesthesiologists, scrub technicians, circulator nurses, or other medical doctors such as gynecologic oncologists, breast surgeons, or urologists.

An AU and an AMP must be present for the initiation of HDR brachytherapy. Members of the treatment team who may be present during the treatment procedure, but whose attendance is not mandatory include dosimetrists, nurses, and therapists. Additional members of the treatment team who may be present for applicator placement and/or treatment delivery, but who are not required, include trainees such as radiation oncology or medical physics residents, and dosimetry or radiation therapy technology students. Treatment should be delivered in compliance with local regulations and facility policies.

### 6.2 Training and competency

The federal and state regulations outline the specific education and training requirements for individuals holding the titles of AMP, AU, and RSO (radiation safety officer). These requirements must be followed even if not articulated in this report as they are beyond the scope of this MPPG. Additionally, individuals involved in an HDR brachytherapy program must hold the appropriate (advanced) degree for their specialty. With a few exceptions, individuals must be board certified by the appropriate specialty board which may include the American Board of Radiology (ABR), American Board of Medical Physics (ABMP), Medical Dosimetry Certification Board (MDCB), or American Registry of Radiology Technologists (ARRT). Team members must be licensed if employed in a state that requires licensure (FL, HI, NY, or TX).

Regulations also outline the minimum expected initial and continuing education requirements for participants involved in an HDR brachytherapy program. Additionally, such participants (not previously described) should participate in emergency training, an emergency response drill, HDR‐specific radiation safety training, and in‐service training on an annual basis. Annual training should be completed on all relevant equipment, including the remote afterloader, applicators, transfer tubes, and the treatment planning software. The workflow for each procedure should be reviewed annually. Vendor‐supplied or vendor‐supported training of the treatment unit and treatment planning system should be performed for all relevant staff involved in the HDR brachytherapy program. This is particularly true for new programs. Additionally, on‐site training for the first few cases of each treatment site should be attended by both the vendor and the treatment team. This includes both a new afterloader facility, or new complex applicators such as multicatheter breast brachytherapy or interstitial brachytherapy. Training must be documented, and the documentation should include the training scope as well as a list of the individuals present.

### 6.3 Credentialing

Credentialing of staff can be complex and involve different departments and agencies. Medical staff is often credentialed when newly hired. Training and licenses are verified as part of the local hospital credentialing office in order to grant hospital privileges. To use radioactive material and be identified as an AU or AMP on a RAM license, credentialing is commonly granted by the Radiation Safety Committee, the NRC, the Agreement State, or a combination of these entities. The radiation oncology department may also have its own workflows in order to credential or deem individuals as competent to participate or perform an HDR brachytherapy procedure independently. In some instances, this may involve proctoring and supervision of a defined number of cases and may be site‐specific. Each health care facility should develop an on‐boarding procedure and associated documentation that includes how an individual will demonstrate knowledge for the different types of procedures performed locally. Each individual should be responsible for reading the policies and procedures of the brachytherapy program, observing and performing a predetermined number of cases under supervision, and demonstrating competency. This on‐boarding process should be documented and maintained by the health care facility. Government‐run health care facilities, such as the Veterans Affairs health system, may have other applicable rules that must be understood and followed by the AMP. Annual refreshers or in‐services as well as annual competency evaluations may be helpful in maintaining proficiency.

**TABLE 4:** HDR brachytherapy afterloader QA with periodicity and tolerance

**Periodic test description**	**Frequency**	**Tolerance recommended (required)**
Source strength measurement	SE	3% (5%)
Source positioning accuracy^I^	SE, D	1 (2) mm
Source retraction with backup battery upon power failure	SE	Functional
Timer accuracy^II^	SE, D	1 s or 1% whichever is greater
Timer linearity^III^	AS	1% (3%)
Electrical interlocks at room entrance (door interlock(s))	SE, D	Functional
Emergency source retraction button	SE, D	Functional
“Last person” out button (if present)	SE, D	Functional
Treatment interrupt button	SE, D	Functional
Source out indicators on the unit, console, wall, and so forth	SE, D	Functional
Audio/visual systems	D	Functional
Emergency response kit complete	D	Functional
Independent radiation room monitor and remote display	D	Functional
Calibrated survey meter present	D	Functional
Console computer date and time accuracy	SE, D, Daylight time changes	1 h
Decayed source strength (or activity) in console (compared to decay chart)	SE, D	1%
Catheter misconnect/channel/turret check	D	Functional
TPS to console software communication	SE	Functional

Abbreviations: AS, after service; D, daily (on patient treatment day); SE, source exchange.

## 7 HARDWARE

### 7.1 Treatment Delivery System QA

Broadly defined as “afterloader QA,” the following sections describe the minimum frequency and tolerances of a variety of tests required to ensure ongoing functionality of the console area, the afterloader, as well as specific tests to be performed during commissioning. Commissioning tests must be performed before beginning clinical treatments and all tests in Table 4 must be performed at this point. All ancillary equipment and accessories such as printers, barometers, clamps, and so forth, must be tested prior to use. In cases where the afterloader console is integrated with patient record and verify systems, that communication must be validated at commissioning as well. An alternative plan transfer method should also be in place in case a network disruption occurs to ensure that patients can be treated timely and correctly. Vendors may perform preventative maintenance inspections (PMIs) on an annual or biannual basis, depending on the manufacturer and service contract. Evidence of the PMI should be maintained. Daily QA must be performed after any repair service to the afterloader. A discussion on appropriate source strength measurement methods also follows in Section 7.2. Items marked with a Roman numeral in the Table are further explained in the sections that follow.

#### 7.1.1 Source positioning accuracy

The NRC required tolerance value of 1 mm may be difficult to achieve in a variety of applications but should be verifiable under a fixed test geometry that is used during source exchange. Since the source must be measured within a TGT that itself can only be measured to an accuracy of 1 mm, a more practical tolerance value may be 2 mm as adopted by the report of TG‐56 and COMP. The overall source position accuracy should be 1 mm and must be 2 mm.

#### 7.1.2 Timer accuracy

The timer on the console computer must be accurate to deliver the intended radiation dose to the patient. The dwell time minimum threshold for various afterloaders may be as low as 0.1 s, which cannot be verified via conventional means. One method to check for gross errors is to use an independent stopwatch or timer and deliver a fixed treatment time (using a reasonable clinical time where disparities due to human reflexes are negligible). The accuracy must be within 1 s or 1% (whichever is greater) under these fixed test conditions.

#### 7.1.3 Timer linearity

The dwell time linearity must be validated over at least three treatment times where transit time (typically 1–2 s) is insignificant. For example, the well chamber reading with a 60‐s dwell must be twice the reading of a 30‐s dwell (to within 3%). If one accounts for and removes the reading due to transit time, the agreement may be closer to 1%. The linearity should not change over time unless the afterloader motor is adjusted for example, at a PMI.

### 7.2 Source strength

The air‐kerma strength of each ^192^Ir source used for HDR treatments must be accurately determined and properly accounted for in each treatment. Upon receipt and installation of a new ^192^Ir HDR source, the air‐kerma strength value will be provided by the manufacturer in a calibration certificate with a specific reference date and time. It is the responsibility of the user to verify this value upon receipt of the source by performing measurements using a calibrated well‐type ionization chamber and electrometer. The well‐chamber determined value must agree with the manufacturer's source certificate's value (both decay corrected to a reference date and time) to within 5% although typical agreements are closer to 3%.^41^ If the measurement is outside of this agreement criteria, investigation into the possible reasons must immediately be pursued. It is recommended to check the reference date, the recorded ambient air conditions (temperature and pressure), and most recent well chamber calibration coefficient before contacting the manufacturer. It is uncommon for the difference to be greater than 5%, so treatments must not proceed until this discrepancy is resolved. Either vendor or institutional value may be used if it is applied consistently. In the United States, the well‐type ionization chamber and electrometer should be calibrated by an Accredited Dosimetry Calibration Laboratory (ADCL) at least once every 2 years with traceability to the National Institute of Standards and Technology (NIST). The well‐chamber must have a holder specific for an HDR source catheter and the same holder must be used for the ADCL calibration as well as the end user's clinical source strength measurement. The maximum reading of a source dwell position inside the well chamber should be determined by means of stepping the source in small increments through the well‐chamber holder to find the position where the highest ionization current is produced. This is commonly referred to as the well chamber sweet spot and is unique to each well chamber and source holder.

### 7.3 Applicators and TGTs

Applicator commissioning and QA rely on a variety of physical, imaging, and radiological tests to ensure positional, temporal, and dose delivery accuracy. In general, these tests are described in the AAPM Report 59.^17^ The tests in Table 5 describe the QA tests that must be performed and refer to multi‐use (i.e., not sterile single‐use) applicators and TGTs. Items marked with a Roman numeral in the Table are further explained in the sections that follow.

#### 7.3.1 Autoradiography

Autoradiography used to be the standard method of confirming the active source positioning within the applicator and validation of any planning off‐sets. However, due to many clinics becoming “film free,” this has become more challenging. Alternatives may still be possible using either C‐arms or Linacs (particularly electron beams) and radiochromic film.^42^ Care should be taken to properly identify the source path and locations within the applicator and any offsets characterized. With the advent of solid applicator libraries, the users may have more confidence in vendor‐provided offsets.

#### 7.3.2 Applicator and TGT length

In general, if applicators are solid metal or plastic and the TGTs are stored properly, the lengths of the applicator and TGT combination will rarely change more than 1 mm. All applicators and tubes that are in clinical rotation should be checked on at least an annual basis and compared to the commissioning baseline. The applicator + TGT length should be checked prior to treatment initiation or at least once prior to a fractionated treatment where the applicators are not removed between fractions. As this is one of the most common HDR errors,^43^ site‐specific recommendations will be given in part B of this guideline.

#### 7.3.3 Source positioning

Certain applicators may be highly sensitive to the positioning of the HDR source, which may change slightly over time and with repeated active runs and/or source exchanges. Examples may include tandem and ring, complex gynecological applicators, conical skin applicators, and some shielded applicators. This may affect the output (for conical applicators) or dose distribution surrounding the applicators when a PMI or source exchange occurs. For these applicators, the IFU regarding QA should be followed and tests should be performed to ensure consistent dose delivery. If determined to be a reasonable approximation, offsets over multiple source exchanges and afterloaders can be averaged and used for clinical use. A good discussion of source positional accuracy may be found in Kirisits et al.^27^

Applicators and TGT combination length measurements must be performed annually while in routine use. A failure mode and effects analysis or similar review could be performed to inform the basis for more practical periodicity for those devices, which are found to not change with time. Part B of this report will discuss patient treatment aspects regarding treatment length for planning purposes. Applicator and transfer tube combinations that have not been used in the past year should be tested prior to clinical use. It is also good practice to annually verify the accuracy of the adjustable length gauge and/or length measurement devices if applicable.

Single‐use or one‐time‐use devices are considerably different in that they are often supplied by the manufacturer sterile and may already be placed in a patient by the time the patient presents for treatment in the facility. The specific patient handling aspects for these devices will be handled in part B of this report. It is recommended that the AMP performs QA and testing with a nonsterile test device prior to clinical implementation. Some applicators come nonsterilized and can be tested prior to sterilization. For patient treatments, the combined length of the applicator and transfer tube must be measured and documented at least once per device.

Manufacturer specifications for end of life should be followed as articulated in the IFU. However, using an applicator beyond its stated end of life may be considered under some circumstances if care is taken to ensure the integrity of the applicator and mechanical functioning. Vendors recuse themselves from liability when equipment is used beyond end‐of‐life recommendations. If the applicator exceeds the number of sterilization cycles, material fatigue and infection control may become an issue.

Homemade applicators (machined or 3D printed) add flexibility and the possibility to customize applicator geometry to the patient. The burden of establishing biocompatibility for the materials used (especially if used interstitially or surgically), cleaning, and sterilization procedures must be determined by the hospital team. Because of the high cost of validating repeated cleaning and sterilization cycles between patients, these applicators are typically single‐use. Applicator design and material selection should also reflect the imaging modality intended to be used for planning and treatment verification. Usually made of a plastic material, these applicators are often MR‐safe. Commissioning and validation of applicator geometry and function must be performed and documented for each applicator, as described above. Further guidance may be provided in the forthcoming report of TG‐336 or other published works on 3D printing applications.^44^

Geometric accuracy of shielded applicators must be verified after applicator assembly. A CT scan should be performed at commissioning to understand applicator geometry with and without shields in place. Dynamic shields must be tested for functionality and reproducibility.^28^ If shielding orientation is marked on the applicator, it should be checked for correctness. Solid applicators and the solid applicator library comparison will be discussed in the treatment planning QA section and in Table 8.

**TABLE 5:** Applicator and transfer guide tube tests with frequency and tolerance

**Test description**	**Frequency**	**Tolerance recommended (required)**
Visual inspection of integrity of applicators, tubes, and connections (used that day) by AU or AMP	D	Pass/Fail
Autoradiography (if possible)^I^	C	Baseline
Length of applicator and TGT combination^II^	A, D (see text), C	1 mm (2 mm)
Connection of source TGTs, applicators, and transfer‐tube interfaces	C	Pass/Fail
Geometric integrity of applicator	C	Pass/Fail
Verification of source path and any offsets	C	Baseline
Confirmation of match between solid applicator library and physical applicator	C	1 mm (2 mm)
Source positioning within certain applicators^III^	As needed	Baseline

Abbreviations: A, annually; C, commissioning; D, daily (on day of use).

## 8 SOFTWARE

### 8.1 Treatment planning imaging and tool QA

Treatment planning software commissioning tasks are designed to ensure that the new software package handles clinical tasks such as image manipulation, structure delineations, and dose calculation correctly and some tests will need to be conducted to provide a baseline for periodic checks such as annual QA. Software used for HDR brachytherapy treatment planning may be dedicated to a specific HDR afterloader. In addition, various software packages exist to accommodate specific brachytherapy procedures. Interfaces with ancillary devices (such as an ultrasound stepper, etc.), configuration, networking, and workflow performance should be tested prior to the clinical use of the software. New commissioning must be performed for each new release of the software in addition to vendor‐required testing. Routine clinical use of the software in an active brachytherapy program will reduce the need for repeated testing as loss of functionality or network connections would be noticed with normal use.

**TABLE 6:** HDR brachytherapy TPS imaging and planning tool validation tests

**Test description**	**Tolerance recommended (required)**	**Required**
Image transfer and reconstruction	Pass/Fail	✓
Patient orientation	Pass/Fail	✓
Labeling	Pass/Fail	✓
Geometric accuracy	Modality‐dependent (see text)	✓
Image registration	Modality‐dependent (see text)	
Contouring	Functional	
Source, point, and line delineation	1 mm (2 mm)	
External device interfaces (e.g., steppers)	Functional	

Imaging systems and treatment applicators used in HDR brachytherapy may result in imaging artifacts or distortion, which may lead to incorrect patient dose.^45^, ^46^ While a full discussion of artifacts is beyond the scope of this report, care should be taken to minimize and understand various imaging limitations. The imaging tests that must be performed (required) for TPS commissioning include the recommendations in Table 6 and are discussed in the text below:

#### 8.1.1 Image transfer

Useability of images and image sets imported and exported from the software including DICOM format and live video acquisition. Images should maintain quality and be free of distortion or degradation.

#### 8.1.2 Orientation

Patient orientation is correctly displayed on images acquired using fixed imagers, mobile imagers, and non‐DICOM image acquisition methods where patient orientation is not included in the image data.

#### 8.1.3 Labeling

Transfer of image data including image identifiers, acquisition parameters, and imager information.

#### 8.1.4 Geometric accuracy

The accuracy of the imaging set depends on the modality of the images. CT image accuracy should be within 1 mm in‐plane^47^ and 2 mm elsewhere while MR should be within 2 mm^48^, ^49^ and ultrasound should be within 2 mm or 2%.^50^

#### 8.1.5 Image registration

Rigid registration is most widely used. Multiple scans of the same phantom in different orientations can be aligned and evaluated. Quantitative errors can be measured in some systems using point‐to‐point matching between imaging sets and evaluating the target registration error. Achievable target registration errors should be in the 2–3‐mm range.^51^ Deformable image registration for brachytherapy is currently an active area of research and the registration and dosimetric errors may be large, for example, when registering an image set without the applicator in situ to an image set containing an applicator.^52^, ^53^

#### 8.1.6 Source, point, and line delineation

Point delineation should be accurate to within 1 mm when compared with DICOM coordinates. Both 2D and 3D structure interpolations and expansions should be checked. Reference lines and reference points can be used as surrogates to structure contours and may be used for dose optimization and evaluation. 3D definition of the line and point coordinates should be verified. Structures may be contoured with the use of Boolean operators that should be verified to be performing correctly.

#### 8.1.7 External device interface

Some dedicated planning and delivery systems offer an option for interfacing with external devices. New devices are continually being developed to enhance the safety and consistency of treatments. Examples include electronic and robotic steppers for prostate implants, navigational devices for spine brachytherapy, and electromagnetic tracking. Functional and operational checks of these devices should be performed but specific QA tests are beyond the scope of this guidance report.

### 8.2 Treatment planning source validation and dose calculation

Prior to TPS commissioning, a qualified medical physicist (QMP) must select the dose computation algorithm(s) to be used clinically. The QMP should have a clear understanding of the algorithm(s) chosen, the source model parameters, and how each option affects the resulting dose distributions. There are a variety of commercial and noncommercial brachytherapy treatment planning systems and a given TPS may include multiple dose calculation algorithms. The AAPM currently recommends using a modified AAPM report of TG‐43 dosimetry formalism for clinical dose calculation as defined in AAPM Report 229^25^(subsequently referred to as Report 229), which uses tabulated data to allow calculation of point doses and 3D dose distributions. The tests for source validation and dose calculation accuracy are provided in Table 7. Model‐based dose calculation algorithms (MBDCAs) are also commercially available and the AAPM report of TG‐186 provides recommendations for commissioning these algorithms.^54^ Practice guidelines for MBDCA commissioning are beyond the scope of this report. Due to possible dosimetric implications on the treatment prescription, MBDCAs should not be used clinically without rigorous validation and substantial brachytherapy experience.

#### 8.2.1 Source model data

Source reference data used by the brachytherapy TPS must be appropriate for the source type used for treatment delivery. It is recommended that consensus datasets from Report 229 are used for dose calculations. When checking source parameters in a TPS, the input data must correspond exactly with the published consensus dataset for that source.

#### 8.2.2 Source decay

Some treatment planning systems allow the user to account for radioactive decay. This should be checked with an independent calculation or other validation method.

#### 8.2.3 Plan normalization, weighting, and scaling

Treatment plans are often improved by adjusting isodose distributions globally or locally. Changing the number of fractions or prescribed dose can also scale the dwell times. Treatment times should be cross‐checked to validate the correct scaling of the planned time.

#### 8.2.4 Dose calculation grid

Brachytherapy treatments often involve small calculation volumes and dose accuracy can depend on the calculation grid size used. A large calculation grid may influence DVH calculations, particularly maximum point doses within a contour. Typically, the dose grid resolution may be set at 0.1–0.3 cm per dimension.

#### 8.2.5 Point dose calculations

Either the source model data or a point dose calculation may be verified on an annual basis as these two tests investigate the same process. Users may wish to create a fixed geometry test plan and compare dosimetry annually. If MBDCAs are to be used, dose consistency with AAPM Report 229 based calculations should be verified as well as inhomogeneity and scatter modeling accuracy.

#### 8.2.6 Dose display

The dose should display in both absolute (Gy) and relative (%) doses. If applicators with shields are to be used, a methodology for documentation and isodose line reduction should be incorporated into the planning guidelines if using the Report 229 formalism.

#### 8.2.7 DVH calculation

According to the report of TG‐53, DVH analysis should be performed at least annually. However, for a brachytherapy TPS, the user is required to validate functionality rather than accuracy. Interested users may use the methodology of Gossman et al.^55^

### 8.3 Miscellaneous commissioning tests

Additional tests for software commissioning that should or must be performed are included in Table 8. Some tests may be vendor‐specific and may not apply to all brachytherapy TPS, in which case the requirement is waived.

#### 8.3.1 Optimization validation

Optimization of HDR brachytherapy treatment plans can occur in several ways, including, but not limited to, manual dwell time or weight adjustments, dose shaper or graphical optimization, geometric optimization, and inverse planning algorithms. Assessment of optimization should occur for each available optimization method and should be completed for each treatment or applicator type in clinical use in the department where appropriate.

#### 8.3.2 TPS output

Treatment plan document verification as well as integrity testing of data transfer from the TPS to treatment unit must be completed.

#### 8.3.3 Applicators and catheters

For applicators with known geometry or applicators with template/solid applicator libraries, visualization and digitization/reconstruction must be verified and should agree to the known geometry within +/−1 mm by superimposing a CT image of the applicator onto the geometrical representation of the applicator. A “solid” applicator refers to a vendor provided geometric and dwell position representation of a particular applicator that may be imported into the planning system. Free hand needle and catheter reconstruction may require image interpolation and rotations.

The TPS may have tools to assist with auto‐segmentation of the source path. These tools should be checked, and their limitations documented. For example, noisy images, crossing of catheters/needles, use of dummy wires, high curvature of the catheters, or use of non‐CT images may impair correct detection of the source path. CT range finders may be used for applicator delineation and should also be evaluated for functionality. Digitized/reconstructed source positions within the applicator should be within +/−2 mm of true source positions. However, this limit may not be appropriate depending on the applicator and modality type used.

#### 8.3.4 Independent dose calculation

In HDR brachytherapy, an independent treatment time calculation has historically been performed to verify that the total dwell times and/or dose distribution is consistent with the specified arrangement including source positions, strength, and dwell times. There are a number of commercial checking programs available, however, these secondary programs rely on DICOM input from the TPS for secondary calculations and typically cannot find planning errors. Software that performs independent dose calculations based on independent implant reconstruction has been reported,^56^ as have script‐based algorithms and other software packages that check for consistency of the plan with prescription, as well as other electronic medical record parameters and quality indices.^57^ The recommendation of this practice guideline is that any secondary dose calculations should be optional as they lack true independence or are otherwise not readily available or practical. Any adopted independent system can be verified using the TG53 methodology (Appendix 5).^28^ They should be used with care to ensure that dose calculation has not been corrupted within the TPS, and only after implant geometry and plan parameters have been independently verified. Other independent treatment time calculations (e.g., nomograms, Manchester and Quimby tables) may also be valuable tools for HDR plan QA. Depending on the type of implant these methods can typically predict a plan total dwell time with an accuracy of 5–10%. A secondary dose calculation is separate from an independent plan check that will be addressed in more detail in part B.

#### 8.3.5 Dry run testing

The brachytherapy team must conduct at least one “dry run” functionality test of the entire brachytherapy process from imaging to dose delivery for each treatment technique. This testing should be performed prior to the implementation of a new treatment type and when a key aspect of any process has been modified. Each step in the process should be performed by the staff member who will perform the step when the program is clinically implemented. The dry run test should involve imaging of the applicator through anticipated mechanism, practical treatment planning, connection to tubes and afterloader, and delivery of planned treatment.

**TABLE 7:** HDR brachytherapy TPS source validation and dose calculation tests

**Test description**	**Frequency**	**Tolerance recommended (required)**	**Required test**
Source model data^I^	C, A^a^	Exact	✓
Source decay (if possible)^II^	C, SE	1%	✓
Plan normalization/weighting/scaling^III^	C	Functional	✓
Dose calculation grid^IV^	C	Functional	
Point dose calculation (single source)^V^	C, A^a^	2% (3%)	✓
Point dose calculation (multisource)^V^	C	3% (5%)	
Dose display (absolute and relative)^VI^	C	Functional	
DVH calculation^VII^	C	Functional	✓

Abbreviations: A, annual; C, commissioning; SE, source exchange.

^a^ Perform either test––see discussion in 8.2.5.

**TABLE 8:** HDR brachytherapy TPS commissioning tests

**Treatment planning test description**	**Test specifics**	**Tolerance recommended (required)**	**Required test**
Optimization validation^I^	Manual dwell time/weight	Functional	
	Dose shaper/graphical optimization	Functional	
	Geometric optimization	Functional	
	Inverse planning	Functional	
TPS output validation^II^	Printer or pdf function	Functional	
	Data transfer integrity	Functional	✓
Applicators and catheters^III^	Solid applicator geometry	1 mm (2 mm)	✓
	Source position	2 mm (3 mm) Depends on applicator and modality	✓
	Shielding	Functional	
Independent calculation^IV^ ^a^	Dose calculation	Functional	
Dry run validation^V^	Basic end‐to‐end testing	Functional	✓

^a^ Optional test.

## 9 CONCLUSIONS

Part A of this MPPG provides recommendations for considerations in designing the infrastructure of a HDR brachytherapy program and minimum standards for QA tests for the required equipment. The recommendations from the experts on this practice guideline are intended to guide adoption of regulations in the future.

## AUTHOR CONTRIBUTION

All authors contributed to the writing of the manuscript.

## CONFLICT OF INTEREST

The Chair of TG 348: MPPG 13.1: HDR Brachytherapy has reviewed the required Conflict of Interest statement on file for each member of TG 348 and determined that disclosure of potential Conflicts of Interest is an adequate management plan. Disclosures of potential Conflicts of Interest for each member of TG348 are found at the close of this document. The members of TG348 listed below attest that they have no potential Conflicts of Interest related to the subject matter or materials presented in this document. Susan Richardson, PhD, Chair; Ivan Buzurovic, PhD; Wesley Culberson, PhD; Claire Dempsey, PhD; Bruce Libby, PhD; Christopher Melhus, PhD; Robin Miller, MS; Samantha Simiele, PhD. The members of TG348 listed below disclose the following potential Conflict(s) of Interest related to subject matter or materials presented in this document. Daniel Scanderbeg, PhD––Varian Medical––speaker/consultant Merit Medical––speaker/consultant; Gil'ad Cohen, MS––Varian Medical––speaker.

## References

[1] International Commission on Radiation Units and Measurements (ICRU) . Dose and Volume Specification for Reporting Intracavitary Brachytherapy in Gynecology . Report 38. 1985.

[2] Hilaris BS . Brachytherapy in cancer of the prostate: an historical perspective. Semin Surg Oncol. 1997;13(6). New York: John Wiley & Sons, Inc. 10.1002/(sici)1098-2388(199711/12)13:6<399::aid-ssu3>3.0.co;2-5 9358586

[3] Scalliet P , Gerbaulet A , Dubray B . HDR versus LDR gynecological brachytherapy revisited. Radiother Oncol. 1993;28(2):118‐126. 8248552 10.1016/0167-8140(93)90003-q

[4] International Atomic Energy Agency . Categorization of Radioactive Sources . Safety Standards Series No. RS‐G‐1.9. IAEA; 2005.

[5] Simmons G . Structural Shielding Design and Evaluation for Medical Use of X‐Rays & Gamma Rays of Energies Up to 10 meV . NCRP Report No. 49. NCRP Publications; 1976: 126. $3.50. (1978): 228‐228.

[6] AAPM . Virtual library. Accessed March 6, 2021. https://www.aapm.org/education/VL/vl.asp?id=1993

[7] Nuclear Regulatory Commission . NRC Regulatory Issue Summary 2005‐23. Clarification of the Physical Presence Requirement during Gamma Stereotactic Radiosurgery Treatments . 2005.

[8] Amano Y . Radiation Protection and Safety of Radiation Sources: International Basic Safety Standards . 2011.

[9] National Council on Radiation Protection and Measurements . Limitation of Exposure to Ionizing Radiation . NCRP Report No. 116. National Council on Radiation Protection and Measurements; 1993.

[10] Horton P , Eaton D . Design and Shielding of Radiotherapy Treatment Facilities. IOP Publishing; 2017.

[11] International Atomic Energy Agency . Radiation Protection in the Design of Radiotherapy Facilities . Technical Reports Series No. 47. IAEA; 2006.

[12] International Atomic Energy Agency . Security of Radioactive Material In Use and Storage and of Associated Facilities . Nuclear Security Series No. 11‐G(Rev.1). 2019.

[13] Series, IAEA Safety Standard . Regulations for the Safe Transport of Radioactive Material. TS; 1996.

[14] Valentin J . Prevention of high‐dose‐rate brachytherapy accidents. ICRP Publication 97. Ann ICRP. 2005; 35(2):1‐51. 10.1016/j.icrp.2005.05.002 16243099

[15] Holt JG . Remote Afterloading Technology . AAPM Report No. 41. MEDICAL PHYSICS‐LANCASTER PA‐. 1993;20:1761. 10.1118/1.596938 8309451

[16] AAPM . Comprehensive QA for radiation oncology. TG‐40. Med Phys. 1994;21(4):581‐618. 8058027 10.1118/1.597316

[17] Nath R , Anderson LL , Meli JA , et al. Code of practice for brachytherapy physics: report of the AAPM Radiation Therapy Committee Task Group No. 56. Med Phys. 1997;24(10):1557‐1598. 9350711 10.1118/1.597966

[18] Kubo HD , Glasgow GP , Pethel TD , et al. High dose‐rate brachytherapy treatment delivery: report of the AAPM Radiation Therapy Committee Task Group No. 59. Med Phys. 1998;25(4):375‐403. 9571605 10.1118/1.598232

[19] Thomadsen BR , Erickson BA , Eifel PJ , et al. A review of safety, quality management, and practice guidelines for high‐dose‐rate brachytherapy: executive summary. Pract Radiat Oncol. 2014;4(2):65‐70. 24890345 10.1016/j.prro.2013.12.005

[20] International Atomic Energy Agency . The Transition from 2D Brachytherapy to 3D High Dose Rate Brachytherapy. International Atomic Energy Agency; 2015.

[21] Freniere N . COMP report: CPQR technical quality control guidelines for brachytherapy remote afterloaders. J Appl Clin Med Phys. 2018;19:39–43. [PMID 29417727]] 10.1002/acm2.12272 PMC5859323 29417727

[22] Steenhuizen J , Harbers M , Hoffmann A , Leeuw AD , Rijnders A , Unipan M . NCS Report 30: Code of Practice for Quality Assurance of Brachytherapy with Ir‐192 Afterloaders . 2018.

[23] ACR, ABS, ASTRO . Practice parameter for the performance of radionuclide‐based high‐dose‐rate brachytherapy. 2020. Accessed: January 30, 2023. https://www.acr.org/‐/media/ACR/Files/Practice‐Parameters/hdr‐brachyro.pdf 10.1016/j.brachy.2021.08.009 34588143

[24] ACR, AAPM . Technical standard for the performance of high‐dose‐rate brachytherapy physics. 2020. Accessed: January 30, 2023. https://www.acr.org/‐/media/ACR/Files/Practice‐Parameters/hdr‐brachyts.pdf

[25] Dosimetry, High Energy Brachytherapy Source . Dose Calculation for Photon‐Emitting Brachytherapy Sources with Average Energy Higher than 50 keV: Full Report of the AAPM and ESTRO . 2012. 10.1118/1.3703892 22559663

[26] DeWerd LA , Ibbott GS , Meigooni AS , et al. A dosimetric uncertainty analysis for photon‐emitting brachytherapy sources: Report of AAPM Task Group No. 138 and GEC‐ESTRO. Med Phys. 2011;38(2):782‐801. 21452716 10.1118/1.3533720 PMC3033879

[27] Kirisits C , Rivard MJ , Baltas D , et al. Review of clinical brachytherapy uncertainties: analysis guidelines of GEC‐ESTRO and the AAPM. Radiother Oncol. 2014;110(1):199‐212. 24299968 10.1016/j.radonc.2013.11.002 PMC3969715

[28] Fraass B , Doppke K , Hunt M , et al. American Association of Physicists in Medicine Radiation Therapy Committee Task Group 53: quality assurance for clinical radiotherapy treatment planning. Med Phys. 1998;25(10):1773‐1829. 9800687 10.1118/1.598373

[29] Villarreal‐Barajas JE . COMP report: CPQR technical quality control guidelines for treatment planning systems. J Appl Clin Med Phys. 2018;19(2):35‐38. 10.1002/acm2.12268 PMC5849816 29377492

[30] Beaulieu L , Carlsson Tedgren A , Carrier JF , et al. Report of the Task Group 186 on model‐based dose calculation methods in brachytherapy beyond the TG‐43 formalism: current status and recommendations for clinical implementation. Med Phys. 2012;39(10):6208‐6236. 23039658 10.1118/1.4747264

[31] Fulkerson RK , Perez‐Calatayud J , Ballester F , et al. Surface brachytherapy: joint report of the AAPM and the GEC‐ESTRO Task Group No. 253. Med Phys. 2020;47(10):e951‐e987. 32862452 10.1002/mp.14436

[32] Valentin J . Prevention of high‐dose‐rate brachytherapy accidents. ICRP Publication 97. Ann ICRP. 2005;35(2):1‐51. 10.1016/j.icrp.2005.05.002 16243099

[33] Huq MS , Fraass BA , Dunscombe PB , et al. The report of Task Group 100 of the AAPM: application of risk analysis methods to radiation therapy quality management. Med Phys. 2016;43(7):4209‐4262. 27370140 10.1118/1.4947547 PMC4985013

[34] ASTRO . Safety is no accident: a framework for quality radiation oncology care. 2019. Accessed January 30, 2023. https://www.astro.org/Patient‐Care‐and‐Research/Patient‐Safety/Safety‐is‐no‐Accident

[35] Glasgow GP , Bourland JD , Grigsby PW , Meli JA , Weaver KA . Remote Afterloading Technology . AAPM Report 41. 1993.

[36] International Atomic Energy Agency . Implementation of High Dose Rate Brachytherapy in Limited Resource Settings. International Atomic Energy Agency; 2015.

[37] Lloyd S , Alektiar KM , Nag S , et al. Intraoperative high‐dose‐rate brachytherapy: an American Brachytherapy Society consensus report. Brachytherapy. 2017;16(3):446‐465. 28173994 10.1016/j.brachy.2017.01.001

[38] Tom MC , Joshi N , Vicini F , et al. The American Brachytherapy Society consensus statement on intraoperative radiation therapy. Brachytherapy. 2019;18(3):242‐257. 31084904 10.1016/j.brachy.2019.01.015

[39] Fields EC , Weiss E . A practical review of magnetic resonance imaging for the evaluation and management of cervical cancer. Radiat Oncol. 2016;11(1):1‐10. 26830954 10.1186/s13014-016-0591-0 PMC4736634

[40] Pötter R , Federico M , Sturdza A , et al. Value of magnetic resonance imaging without or with applicator in place for target definition in cervix cancer brachytherapy. Int J Radiat Oncol Biol Phys. 2016;94(3):588‐597. 26867887 10.1016/j.ijrobp.2015.09.023

[41] Vijande J , Carlsson Tedgren Å , Ballester F , et al. Source strength determination in iridium‐192 and cobalt‐60 brachytherapy: a European survey on the level of agreement between clinical measurements and manufacturer certificates. Phys Imaging Radiat Oncol. 2021;19:108‐111. 34401536 10.1016/j.phro.2021.07.007 PMC8348214

[42] Evans MDC , Devic S , Podgorsak EB . High dose‐rate brachytherapy source position quality assurance using radiochromic film. Med Dosim. 2007;32(1):13‐15. 17317530 10.1016/j.meddos.2006.10.001

[43] Richardson S . A 2‐year review of recent Nuclear Regulatory Commission events: what errors occur in the modern brachytherapy era? Pract Radiat Oncol. 2012;2(3):157‐163. 24674119 10.1016/j.prro.2011.08.004

[44] Ricotti R , Vavassori A , Bazani A , et al. 3D‐printed applicators for high dose rate brachytherapy: dosimetric assessment at different infill percentage. Phys Med. 2016;32(12):1698‐1706. 27592531 10.1016/j.ejmp.2016.08.016

[45] Schmid M , Crook JM , Batchelar D , et al. A phantom study to assess accuracy of needle identification in real‐time planning of ultrasound‐guided high‐dose‐rate prostate implants. Brachytherapy. 2013;12(1):56‐64. 22513104 10.1016/j.brachy.2012.03.002

[46] Kim Y , Muruganandham M , Modrick JM , et al. Evaluation of artifacts and distortions of titanium applicators on 3.0‐Tesla MRI: feasibility of titanium applicators in MRI‐guided brachytherapy for gynecological cancer. Int J Radiat Oncol Biol Phys. 2011;80(3):947‐955. 20934275 10.1016/j.ijrobp.2010.07.1981

[47] Mutic S , Palta JR , Butker EK , et al. Quality assurance for computed‐tomography simulators and the computed‐tomography‐simulation process: report of the AAPM Radiation Therapy Committee Task Group No. 66. Med Phys. 2003;30(10):2762‐2792. 14596315 10.1118/1.1609271

[48] Ranta I , Kemppainen R , Keyriläinen J , et al. Quality assurance measurements of geometric accuracy for magnetic resonance imaging‐based radiotherapy treatment planning. Phys Med. 2019;62:47‐52. 31153398 10.1016/j.ejmp.2019.04.022

[49] Kavaluus H , Nousiainen K , Kaijaluoto S , et al. Determination of acceptance criteria for geometric accuracy of magnetic resonance imaging scanners used in radiotherapy planning. Phys Imaging Radiat Oncol. 2021;17:58‐64. 33898780 10.1016/j.phro.2021.01.003 PMC8058029

[50] Pfeiffer D , Sutlief S , Feng W , et al. AAPM Task Group 128: quality assurance tests for prostate brachytherapy ultrasound systems. Med Phys. 2008;35(12):5471‐5489. 19175107 10.1118/1.3006337

[51] Brock KK , Mutic S , McNutt TR , et al. Use of image registration and fusion algorithms and techniques in radiotherapy: report of the AAPM Radiation Therapy Committee Task Group No. 132. Med Phys. 2017;44(7):e43‐e76. 28376237 10.1002/mp.12256

[52] Swamidas J , Kirisits C , De Brabandere M , et al. Image registration, contour propagation and dose accumulation of external beam and brachytherapy in gynecological radiotherapy. Radiother Oncol. 2020;143:1‐11. 31564555 10.1016/j.radonc.2019.08.023

[53] Tait LM , Hoffman D , Benedict S , et al. The use of MRI deformable image registration for CT‐based brachytherapy in locally advanced cervical cancer. Brachytherapy. 2016;15(3):333‐340. 26882845 10.1016/j.brachy.2016.01.002

[54] Beaulieu L , Carlsson Tedgren A , Carrier JF , et al. Report of the Task Group 186 on model‐based dose calculation methods in brachytherapy beyond the TG‐43 formalism: current status and recommendations for clinical implementation. Med Phys. 2012;39(10):6208‐6236. 23039658 10.1118/1.4747264

[55] Gossman MS , Hancock SS , Kudchadker RJ , et al. Brachytherapy dose‐volume histogram commissioning with multiple planning systems. J Appl Clin Med Phys. 2014;15(2):110‐120. 10.1120/jacmp.v15i2.4620 PMC5875493 24710449

[56] Gil'ad NC , Amols HI , Zaider M . An independent dose‐to‐point calculation program for the verification of high‐dose‐rate brachytherapy treatment planning. Int J Radiat Oncol Biol Phys. 2000;48(4):1251‐1258. 11072186 10.1016/s0360-3016(00)00725-2

[57] Simiele SJ , Chen X , Lee C , et al. Development and comprehensive commissioning of an automated brachytherapy plan checker. Brachytherapy. 2020;19(3):355‐361. 32249182 10.1016/j.brachy.2020.02.003

